# Mucosal boosting of chimpanzee adenovirus-vectored vaccine encoding SARS-CoV-2 RBD-heterodimer provides robust protection in mice

**DOI:** 10.1128/mbio.01464-26

**Published:** 2026-07-21

**Authors:** Xueyuan Liu, Yaling An, Huixin Duan, Fei Shao, Ke Xu, Chenxi Yang, Senyu Xu, Xuemei Zhou, Minrun Duan, Tianyi Zheng, Shiyu Sun, Mengjie Yang, Kun Cai, William J. Liu, Dongming Zhou, Kun Xu, Lianpan Dai, Shuo Wang, Peipei Liu, Wei Ma, George F. Gao

**Affiliations:** 1 School of Public Health, Cheeloo College of Medicine, Shandong University, Jinan, Shandong Province, China; 2 CAS Key Laboratory of Pathogen Microbiology and Immunology, Institute of Microbiology, Chinese Academy of Sciences (CAS), Beijing, China; 3 NHC Key Laboratory of Biosafety, National Institute for Viral Disease Control and Prevention, Chinese Center for Disease Control and Prevention (China CDC), Beijing, China; 4 Medical School, University of Chinese Academy of Sciences, Beijing, China; 5 College of Animal Science and Veterinary Medicine, Guangxi University 12664, Nanning, China; 6 Zhejiang University School of Medicine 26441, Hangzhou, China; 7 Strategic Pandemic Preparedness Action and Research Center (SPARC), Guangzhou National Laboratory 612039, Guangzhou, China; 8 Department of Pathogen Biology, School of Basic Medical Sciences, Medical University, Tianjin, China; 9 Institute of Zoology, Chinese Academy of Sciences 53024, Beijing, China; Tsinghua University, Beijing, China

**Keywords:** adenovirus-vectored vaccine, mucosal immune responses, aerosol inhalation, memory T cells

## Abstract

The major route of COVID-19 vaccination currently is via intramuscular injection. Data from clinical trials and real-world studies have demonstrated its effectiveness in preventing severe illness and death caused by SARS-CoV-2 infection. However, its protective efficacy against SARS-CoV-2 infection and transmission *in situ* remains relatively low. Given that SARS-CoV-2, especially the Omicron variant and its sub-variants, primarily infects and replicates in the human upper respiratory tract, mucosal immune responses are crucial for preventing viral infection. Therefore, we constructed a chimpanzee adenovirus (AdC68)-vectored vaccine expressing the Delta-XBB receptor-binding domain (RBD)-dimer and comprehensively compared the immune responses induced by intramuscular injection, intranasal administration, or aerosol inhalation. Our results revealed that aerosol inhalation of the recombinant AdC68 vaccine induced robust systemic and mucosal immune responses and immune memory, particularly activating memory T cells in the lungs with a long duration in the mouse model. Additionally, we assessed long-term protection against a SARS-CoV-2 XBB.1 challenge after ~6 months following a booster vaccination with AdC68-Delta-XBB via different immunization routes. We found that, compared with the intramuscular route, aerosol inhalation provided significantly better protection, without detectable replicating virus in the nasal tissue. This study demonstrates that the AdC68-Delta-XBB vaccine induces robust mucosal immune responses via aerosol inhalation vaccination and prevents SARS-CoV-2 infection in mucosa.

**IMPORTANCE:**

Immunity induced by first-generation COVID-19 vaccines administered by intramuscular injection is highly effective against severe disease and death but is limited in its ability to prevent viral infection and transmission. A more cost-effective and practical vaccine delivered by the respiratory route is needed to better understand mucosal immune responses and to assess protective efficacy. This study evaluated the immune responses and protective efficacy elicited by intramuscular injection, intranasal administration, or aerosol inhalation of AdC68-Delta-XBB in a mouse model and demonstrated that the aerosol inhalation approach is particularly advantageous for robustly stimulating both systemic and mucosal immune responses. These findings will help guide future clinical development and provide a basis for developing vaccines against other respiratory pathogens.

## INTRODUCTION

The COVID-19 pandemic has caused significant global disruption (1, 2). To combat COVID-19, various vaccines have been authorized for large-scale use, including mRNA vaccines, inactivated virus vaccines, protein subunit vaccines, and adenovirus-vectored vaccines (3–5). Data from clinical trials and real-world studies on most COVID-19 vaccines indicate theirhigh efficacy in protecting againstsevere disease and death in preventingthe prototypeof the virus, but their efficacy in preventing viral infection and transmission is limited, especially after the emergence of variants (6–9). The Omicron variant and its sub-variants, especially the XBB and BA.2.86, can efficiently replicate in the human upper respiratory tract and cause symptoms, highlighting the importance of developing COVID-19 vaccines that stimulate mucosal immunity (10–13). Respiratory mucosal immunity research is a spotlight field for respiratory pathogen vaccine development (14–16).

Currently, five COVID-19 vaccines have been approved for use via intranasal spray or aerosol inhalation (17). The human adenovirus type 5-vectored vaccine Convidecia Air (encoding the full-length spike [S] protein) was produced by CanSino and administered through aerosol inhalation (18–22). The other four vaccines are all administered via nasal spray, including the influenza virus-vectored vaccine dNS1-RBD (encoding S protein receptor-binding domain [RBD]) produced by Wantai BioPharm (23, 24), the adenovirus-vectored vaccine BBV154 (encoding S-2P) produced by Bharat Biotech International Limited (25, 26), the adenovirus-vectored vaccine Sputnik V (encoding S) produced by the Gamaleya Research Institute of Epidemiology and Microbiology (27), and the recombinant S protein vaccine RAZI-COV-PARS produced by the Razi Vaccine and Serum Research Institute (28–30). Aerosol inhalation and intranasal spray are two common routes to induce mucosal immune responses. However, the efficacy of these vaccines in protecting humans against SARS-CoV-2 infection has not been studied. Several studies have investigated the protective efficacy of COVID-19 vaccines against viral infections in animal models, such as delivering aerosolized vaccines into the trachea, intranasal drop administration, or dry powder aerosol immunization (31–35). These models are different from aerosol inhalation and nasal spray immunization in humans. In mouse models, vaccine droplets are administered intranasally, allowing the vaccine to reach the lungs, whereas human nasal spray vaccines primarily target the upper respiratory tract. Therefore, a more cost-effective and practical animal model is needed for a better understanding of the mucosal immune responses and protection efficacy of different vaccination routes.

Previously, we developed a protein subunit vaccine, ZF2001, which has been approved in China and several other countries since March 2021, with over 350 million doses administered (36–39). The immunogen of this vaccine is a rationally designed dimeric RBD, eliciting robust neutralizing antibody responses (40, 41). Because protein vaccines stimulate limited T cell responses, we designed a chimpanzee adenovirus (AdC7)-vectored vaccine expressing the RBD-dimer to enhance T cell immunity (42), in addition to the mRNA vaccine (43). This adenovirus vaccine can induce mucosal immune responses following intranasal administration (42). Following the emergence and circulation of SARS-CoV-2 variants and sub-variants, we further developed a bivalently heterologous RBD-dimer to induce broader immune responses (44, 45).

In this study, we further extended a recombinant chimpanzee adenovirus (AdC68)-vectored vaccine (42) expressing the Delta-XBB RBD-heterodimer immunogen and immunized mice via intramuscular injection, intranasal drop, or aerosol inhalation. We then evaluated and compared the induced humoral, cellular, and mucosal immune responses, memory immune responses, as well as the protective efficacy against SARS-CoV-2 challenge. The findings from this study will help guide future clinical development and provide a basis for developing vaccines against other respiratory pathogens.

## RESULTS

### Antibody responses induced by AdC68-Delta-XBB

We constructed and purified a chimpanzee adenovirus (AdC68)-vectored vaccine encoding the SARS-CoV-2 Delta-XBB RBD-dimer immunogen (44, 46), based on our previous AdC7-vectored design (42). The antigen protein expression was demonstrated by western blot in AdC68-Delta-XBB vaccine-infected HEK-293T cells (Fig. S1). Next, we assessed the inhaled dose of aerosolized AdC68-Delta-XBB vaccine in a BALB/c mouse model. One group of mice received an intranasal drip dose of 2.5 × 10^10^ AdC68 viral particles (vp) as a control. The other three groups inhaled aerosolized vaccine at doses of 2.5 × 10^10^, 2.5 × 10^11^, and 2.5 × 10^12^ vp, respectively. Levels of adenovirus genomic DNA (gDNA) from mixed murine lungs, trachea, and nasal turbinate tissues at 2 h after vaccination were measured (Fig. S2). The results suggested that vaccine uptake in mice was comparable between the inhalation group (IH) of 2.5 × 10^12^ vp dose and the intranasal group (IN) of 2.5 × 10^10^ vp dose (Fig. S2). Therefore, we used a 2.5 × 10^12^ vp dose for subsequent aerosol inhalation immunizations.

**Fig 1:**
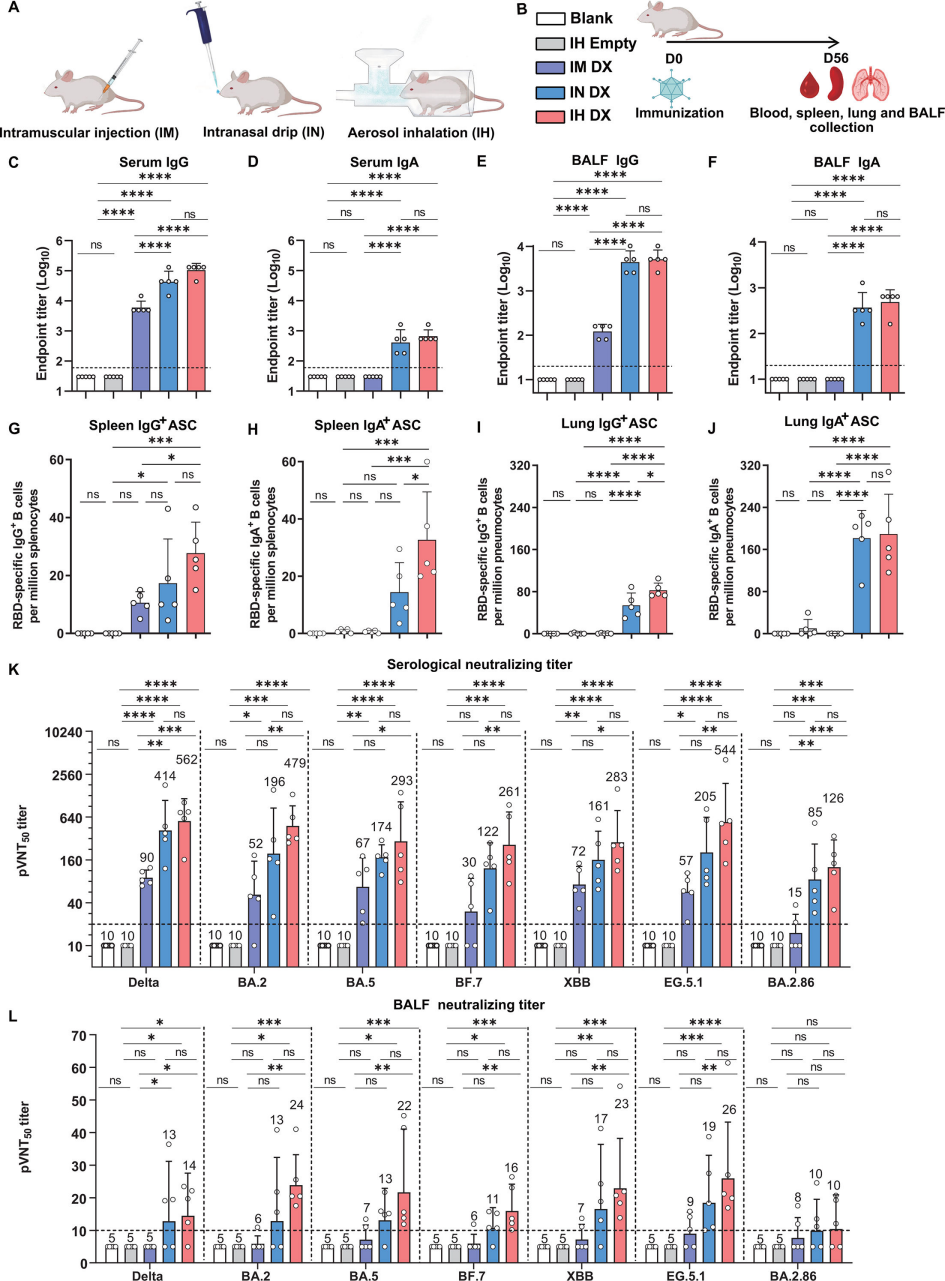
Antibody responses induced by AdC68-Delta-XBB. (**A**) Immunization routes: intramuscular injection (IM), intranasal drip (IN), and aerosol inhalation (IH). (**B**) Experimental schedule. Six- to eight-week-old female BALB/c mice (*n* = 5) were immunized via intramuscular injection, intranasal drip, or aerosol inhalation with AdC68-Delta-XBB. One group of mice was immunized with AdC68-Empty through aerosol inhalation as a control. Another control group did not receive any treatment. Blood, spleen, lung, and BALF were collected at day 56. (**C and D**) Anti-Delta-XBB RBD-dimer IgG (**C**) and IgA (**D**) titers in sera were evaluated by ELISA. (**E and F**) Anti-Delta-XBB RBD-dimer IgG (**E**) and IgA (**F**) titers in BALF were evaluated by ELISA. The values shown in panels **C–F** are the geometric mean titers (GMTs) ± SD. The horizontal dashed line indicates the limit of detection (LOD). The endpoint titer below the LOD was determined as half the LOD. Log-transformed data were statistically analyzed using ordinary one-way ANOVA, followed by Tukey’s multiple comparisons tests (ns, *P* > 0.05; ****P < 0.0001). (**G and H**) Delta-XBB RBD-dimer-specific IgG^+^ (**G**) and IgA^+^ (**H**) ASC frequencies in splenocytes were measured by enzyme-linked immunospot (ELISpots). (**I and J**) Delta-XBB RBD-dimer-specific IgG^+^ (**I**) and IgA^+^ (**J**) antibody-secreting cell (ASC) frequencies in pneumocytes were measured by ELISpots. The values shown in panels **G-J** are mean ± SD. Data were statistically analyzed using ordinary one-way ANOVA, followed by Tukey’s multiple comparisons tests (ns, *P* > 0.05; *P < 0.05; ***P < 0.001; ****P < 0.0001). (**K and L**) Neutralizing antibody titers against SARS-CoV-2 Delta, Omicron BA.2, BA.5, BF.7, XBB, EG.5.1, and BA.2.86 pseudoviruses in murine sera (**K**) and BALF (**L**). Data are GMTs ± SD. Shown at the top of each column are 50% pseudovirus neutralization titers (pVNT_50_). The dashed horizontal line indicates the LOD. pVNT_50_ below the LOD was determined as half the LOD. Log-transformed data were statistically analyzed using ordinary one-way ANOVA, followed by Tukey’s multiple comparisons tests (ns, *P* > 0.05; *P < 0.05; **P < 0.01; ***P < 0.001; ****P < 0.0001).

To analyze and compare the immune responses induced by various administration routes, we immunized groups of BALB/c mice with a single dose of AdC68-Delta-XBB (DX) via intramuscular injection (IM), intranasal drip, or aerosol inhalation (Fig. 1A). A control group was immunized with an empty AdC68 vector vaccine via aerosol inhalation. Another control group did not receive any treatment. On day 56 post-immunization, we collected blood, bronchoalveolar lavage fluid (BALF), spleen, and lung samples for measurement of systemic and mucosal immune responses (Fig. 1B). Blood and BALF were measured for binding (IgG and IgA) and neutralizing antibodies. Spleen and lung tissues were homogenized into single-cell suspensions to analyze systemic and mucosal B- and T-cell responses.

The results indicated that intramuscular immunization induced the production of serological Delta-XBB RBD-dimer-binding IgG antibodies but not IgA antibodies (Fig. 1C and D). Intranasal and aerosol inhalation immunization stimulated significantly higher titers of both IgG and IgA antibodies in sera than intramuscular injection (Fig. 1C and D). In the intramuscular group, Delta-XBB RBD-dimer-specific IgG antibodies were detected in BALF at a geometric mean titer (GMT) of 121, whereas IgA antibodies were undetectable (Fig. 1E and F). In contrast, both the intranasal and aerosol inhalation groups produced higher levels of IgG antibodies with the GMTs of 4457 and 5120, respectively, and IgA antibodies with the GMTs of 368 and 485, respectively (Fig. 1E and F).

We also performed enzyme-linked immunospot (ELISpot) assays to analyze Delta-XBB RBD-dimer-specific antibody-secreting cells (ASCs) in splenocytes and pneumocytes. The results showed that the average number of specific IgG ASCs was 10.6 per million splenocytes in the intramuscular group, compared to 17.3 and 27.7 in the intranasal and aerosol inhalation groups, respectively (Fig. 1G). However, antigen-specific IgA ASCs were almost undetectable in the splenocytes of the intramuscular group, while the intranasal and aerosol inhalation groups had averages of 14.4 and 32.7 IgA ASCs per million splenocytes, respectively (Fig. 1H). In pneumocytes, neither specific IgG nor IgA ASCs were detected in the intramuscular group (Fig. 1I and J). In comparison, the intranasal and aerosol inhalation groups exhibited significantly higher specific IgG ASCs (averages of 53.9 and 82.7 per million pneumocytes, respectively) and IgA ASCs (averages of 181.5 and 189.5 per million pneumocytes, respectively) (Fig. 1I and J). These results indicated that intranasal drip and aerosol inhalation strongly stimulated both systemic and mucosal responses, but not the intramuscular injection.

Next, we measured and analyzed the GMTs of 50% pseudovirus neutralization titers (pVNT_50_) in murine sera. The aerosol inhalation group exhibited the highest serological pVNT_50_ among all groups, inducing a broad neutralizing profile with GMTs ranging from 126 to 562 against pseudoviruses of SARS-CoV-2 Delta, Omicron BA.2, BA.5, BF.7, XBB, EG.5.1, and BA.2.86 (Fig. 1K). The GMTs induced by intranasal drip were lower than those induced by aerosol inhalation, with decreases ranging from 1.36- to 2.65-fold against these tested pseudoviruses (Fig. 1K). In comparison, the intramuscular group showed the lowest GMTs, ranging from 30 to 90 against SARS-CoV-2 Delta, Omicron BA.2, BA.5, BF.7, XBB, and EG.5.1. The pVNT_50_ against BA.2.86 was 15, with most sample titers falling below the limit of detection (LOD) (Fig. 1K).

In addition, pVNT_50_ in BALF was measured (Fig. 1L). Both the aerosol inhalation and intranasal groups exhibited measurable GMTs slightly above the LOD, with no significant difference observed between the two groups (Fig. 1L). These data suggest that a single dose of aerosol inhalation immunization stimulated both systemic and mucosal antibody responses.

### Cellular immune responses in the lung induced by AdC68-Delta-XBB

**Fig 2:**
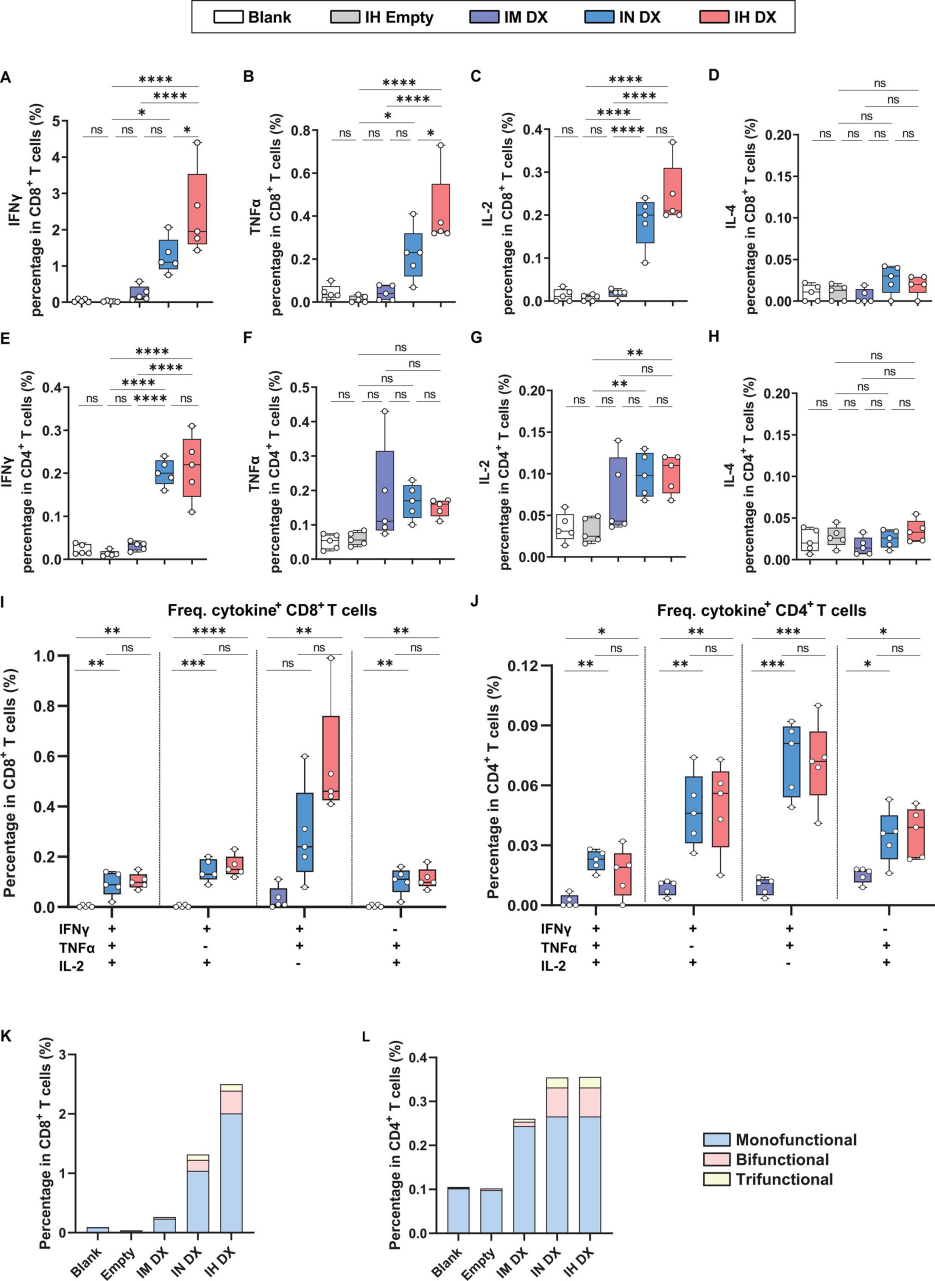
Cellular immune responses in the lung induced by AdC68-Delta-XBB. Evaluation of the cellular immune responses by intracellular cytokine staining. Lungs of mice were collected 56 days post-immunization, followed by *ex vivo* stimulation with mixed overlapping peptide pools of SARS-CoV-2 Delta RBD and XBB RBD. (**A–D**) Quantification of the frequencies of IFNγ (**A**), TNFα (**B**), IL-2 (**C**), and IL-4 (**D**) producing in CD8^+^ T cells of pneumocytes. (**E–H**) Quantification of the frequencies of IFNγ (**E**), TNFα (**F**), IL-2 (**G**), and IL-4 (**H**) producing in CD4^+^ T cells of pneumocytes. (**I and J**) Box-plot graphs depicting multifunctional CD8^+^ (**I**) and CD4^+^ (**J**) T-cell responses in the lung as measured by the production of IFNγ, TNFα, and/or IL-2. (**K and L**) Stacked bar charts showing the functionality of CD8^+^ (**K**) and CD4^+^ (**L**) T cells, defined by the production of IFNγ, TNFα, and IL-2. Data represent the percentages of monofunctional, bifunctional, and trifunctional cells within the gated cytokine-producing T cell populations in lung tissue. For panels **A–J**, shown are the box-and-whiskers plots of the 25th–75th percentiles with the median as the center and whiskers indicating the minimum to maximum values. Data were statistically analyzed using ordinary one-way ANOVA, followed by Tukey’s multiple comparisons tests (ns, *P* > 0.05; *P < 0.05; **P < 0.01; ***P < 0.001; ****P < 0.0001).

To analyze the T cell response induced by the AdC68-Delta-XBB vaccine, murine pneumocytes were isolated and stimulated with SARS-CoV-2 mixed Delta and XBB RBD peptide pools. The results revealed that intranasal drip and aerosol inhalation strongly triggered CD8^+^ T cell responses, with the secretion of IFNγ, TNFα, and IL-2, indicating a Th1-prone cellular immune response (Fig. 2A through D). Especially, the aerosol inhalation group exhibited a significantly higher activation of IFNγ response in CD8^+^ T cells (median, 1.95%) compared to the intranasal (median, 1.10%) or intramuscular (median, 0.15%) groups (Fig. 2A). The aerosol inhalation group also showed a significantly higher percentage of CD8^+^ TNFα^+^ T cells (median, 0.33%) than the intranasal (median, 0.23%) or intramuscular (median, 0.04%) groups (Fig. 2B). Additionally, the aerosol inhalation and intranasal drip induced medians of 0.21% and 0.20%, respectively, IL-2-secreting CD8^+^ T cells, higher than intramuscular injection (median, 0.02%) (Fig. 2C). All three immunization routes did not elicit detectable IL-4-secreting CD8^+^ T cells (Fig. 2D).

For CD4^+^ T cells, intranasal and aerosol inhalation induced IFNγ cellular responses, with percentages (medians of 0.22% and 0.20%, respectively) significantly higher than those in the intramuscular group (median, 0.04%) (Fig. 2E). The percentages of TNFα- and IL-2-secreting CD4^+^ T cells were similar across all three groups vaccinated with AdC68-Delta-XBB (Fig. 2F and G). In addition, none of these three vaccination routes significantly induced IL-4 responses in CD4^+^ T cells compared to the blank and AdC68-Empty control groups (Fig. 2H).

The overall magnitude of antigen-specific T-cell responses in the lungs was measured as frequencies (Fig. 2A through H; Fig. S4 and S5). Given the potent activation of IFNγ-, TNFα-, and IL-2-secreting T cells, we further analyzed the percentage of T cells secreting two or three of these cytokines simultaneously. The results indicated that aerosol inhalation and intranasal drip induced significantly higher percentages of CD8^+^ and CD4^+^ T cells secreting three cytokines than the intramuscular group (Fig. 2I and J). In addition, the highest proportion of multifunctional T cells in intranasal and aerosol inhalation groups was found to be IFNγ^+^ TNFα^+^ CD8^+^ T cells, with median percentages of 0.24% and 0.46%, respectively (Fig. 2I).

We next assessed the functional composition of these responses (Fig. 2K and L; Fig. S4). Although the overall presence of antigen-specific T cells in the lung remained limited in the intramuscular group (Fig. 2A through D), this group exhibited a pronounced skewing toward monofunctional responses. Specifically, the proportion of polyfunctional CD8^+^ and CD4^+^ T cells (secreting two or three cytokines) was higher in the intranasal administration and aerosol inhalation groups than in the intramuscular group (Fig. 2K and L).

These findings demonstrated that intramuscular immunization induced only a minimal frequency of antigen-specific T cells in lung tissue, and aerosol inhalation and intranasal administration robustly induced multifunctional cellular immune responses in the lungs.

### Memory T-cell responses in the lung induced by AdC68-Delta-XBB

Central memory T cells (TCMs), effector memory T cells (TEMs), and tissue-resident memory T cells (TRMs) are subsets of memory T cells with distinct roles and locations in the immune system. TCMs can circulate in lymph nodes and provide long-term immunity by rapidly proliferating and differentiating into effector T cells upon re-exposure to an antigen. TEMs are primarily in peripheral tissues and respond quickly to antigen re-exposure, producing cytokines and carrying out effector functions such as cytotoxicity. TRMs are stationed permanently in non-lymphoid tissues, such as the skin, lungs, or mucosal surfaces, and do not recirculate. They provide rapid and local immune responses to infections where they reside and infections commonly occur (47–50).

**Fig 3:**
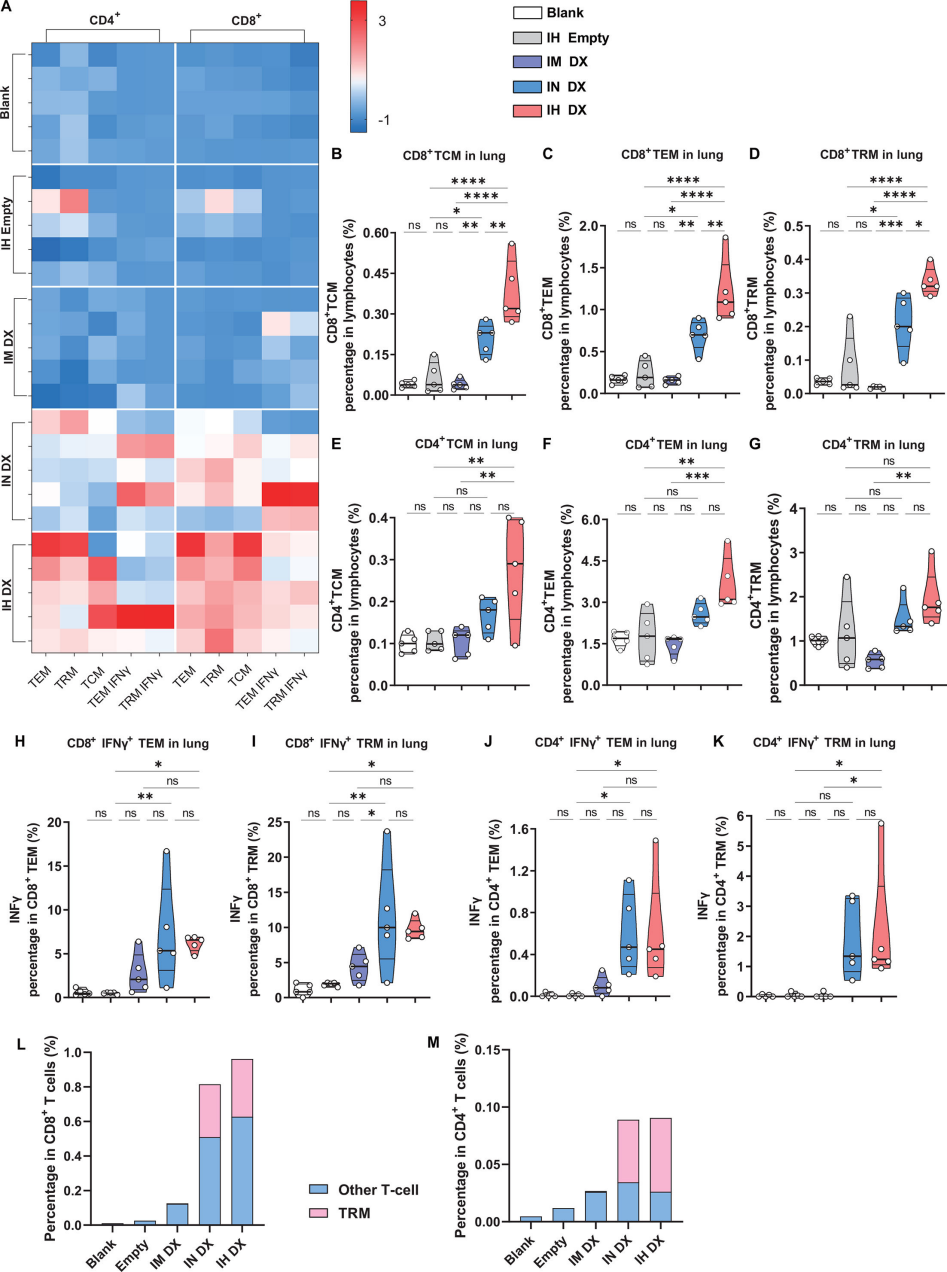
Memory T-cell responses in the lung induced by AdC68-Delta-XBB. (**A**) Heat map showing the levels of TEMs, TRMs, TCMs, IFNγ-secreting TEMs, and IFNγ-secreting TRMs. (**B–G**) The pneumocytes without peptide re-stimulation were characterized by flow cytometry. The frequencies of memory T cells were analyzed by cell surface markers CD8^+^ CD44^+^ CD62L^+^ (CD8^+^ TCMs) (**B**), CD8^+^ CD44^+^ CD62L^−^ (CD8^+^ TEMs) (**C**), CD8^+^ CD44^+^ CD62L^−^ CD69^+^ (CD8^+^ TRMs) (**D**), CD4^+^ CD44^+^ CD62L^+^ (CD4^+^ TCMs) (**E**), CD4^+^ CD44^+^ CD62L^−^ (CD4^+^ TEMs) (**F**), and CD4^+^ CD44^+^ CD62L^−^ CD69^+^ (CD4^+^ TRMs) (**G**). (**H–K**) The pneumocytes were re-stimulated with the mixed overlapping peptide pools for Delta RBD and XBB RBD, and characterized by flow cytometry as CD8^+^ CD44^+^ CD62L^−^ IFNγ^+^ (CD8^+^ IFNγ^+^ TEMs) (**H**), CD8^+^ CD44^+^ CD62L^−^ CD69^+^ IFNγ^+^ (CD8^+^ IFNγ^+^ TRMs) (**I**), CD4^+^ CD44^+^ CD62L^−^ IFNγ^+^ (CD4^+^ IFNγ^+^ TEMs) (**J**), and CD4^+^ CD44^+^ CD62L^−^ CD69^+^ IFNγ^+^ (CD4^+^ IFNγ^+^ TRMs) (**K**). (**L and M**) Stacked bar charts showing the frequencies of IFNγ-producing TRMs and non-TRM IFNγ-producing T cells as percentages of total CD8^+^ (**L**) or CD4^+^ (**M**) T cells in lung tissue. For panels **B–K**, shown are violin plots of the 25th–75th percentiles with the median as the center, and kernel density estimation curves of the distribution. Data were statistically analyzed using ordinary one-way ANOVA, followed by Tukey’s multiple comparisons tests (ns, *P* > 0.05; *P < 0.05; **P < 0.01; ***P < 0.001; ****P < 0.0001).

Our results revealed the disparities in lung memory T cell responses across these three immunization routes (Fig. 3A; Fig. S6). Intramuscular injection did not significantly induce memory T-cell responses in the lungs (Fig. 3B through G). In contrast, intranasal immunization significantly induced the production of CD8^+^ TCMs, TEMs, and TRMs (Fig. 3B through D). Aerosol inhalation elicited significantly higher percentages of both CD8^+^ and CD4^+^ TCMs, TEMs, and TRMs than either the intramuscular or intranasal groups (Fig. 3B through G).

Next, we stimulated pneumocytes with mixed Delta and XBB RBD peptide pools and analyzed IFNγ-secreting memory T cells. The results showed that the intramuscular group had slightly increased percentages of IFNγ-secreting CD8^+^ TEMs (median, 2.06%), CD8^+^ TRMs (median, 4.44%), and CD4^+^ TEMs (median, 0.08%) compared to the blank control group (medians of 0.47%, 0.83%, and 0.0%, respectively) (Fig. 3H through J). In contrast, both the intranasal and aerosol inhalation groups induced high percentages of CD8^+^ IFNγ-secreting TEMs (medians of 5.35% and 6.57%, respectively) and TRMs (medians of 9.98% and 9.42%, respectively) (Fig. 3H and I). In addition, intranasal and aerosol inhalation immunization also induced the IFNγ-secreting TEMs (medians of 0.47% and 0.45%, respectively) and TRMs (medians of 1.34% and 1.24%, respectively) in CD4^+^ cells (Fig. 3J and K). Next, we analyzed the composition of antigen-specific IFNγ^+^ T-cell populations by determining the percentage of TRMs within these populations (Fig. 3L and M). The results revealed that the intranasal administration and aerosol inhalation groups exhibited high TRM phenotype proportion in the pneumocyte IFNγ^+^ T-cell populations (Fig. 3L and M; Fig. S5 and S6).

These results suggested that aerosol inhalation and intranasal drip potently elicit memory T-cell responses in murine lungs.

### Long-term immune responses induced by AdC68-Delta-XBB

**Fig 4:**
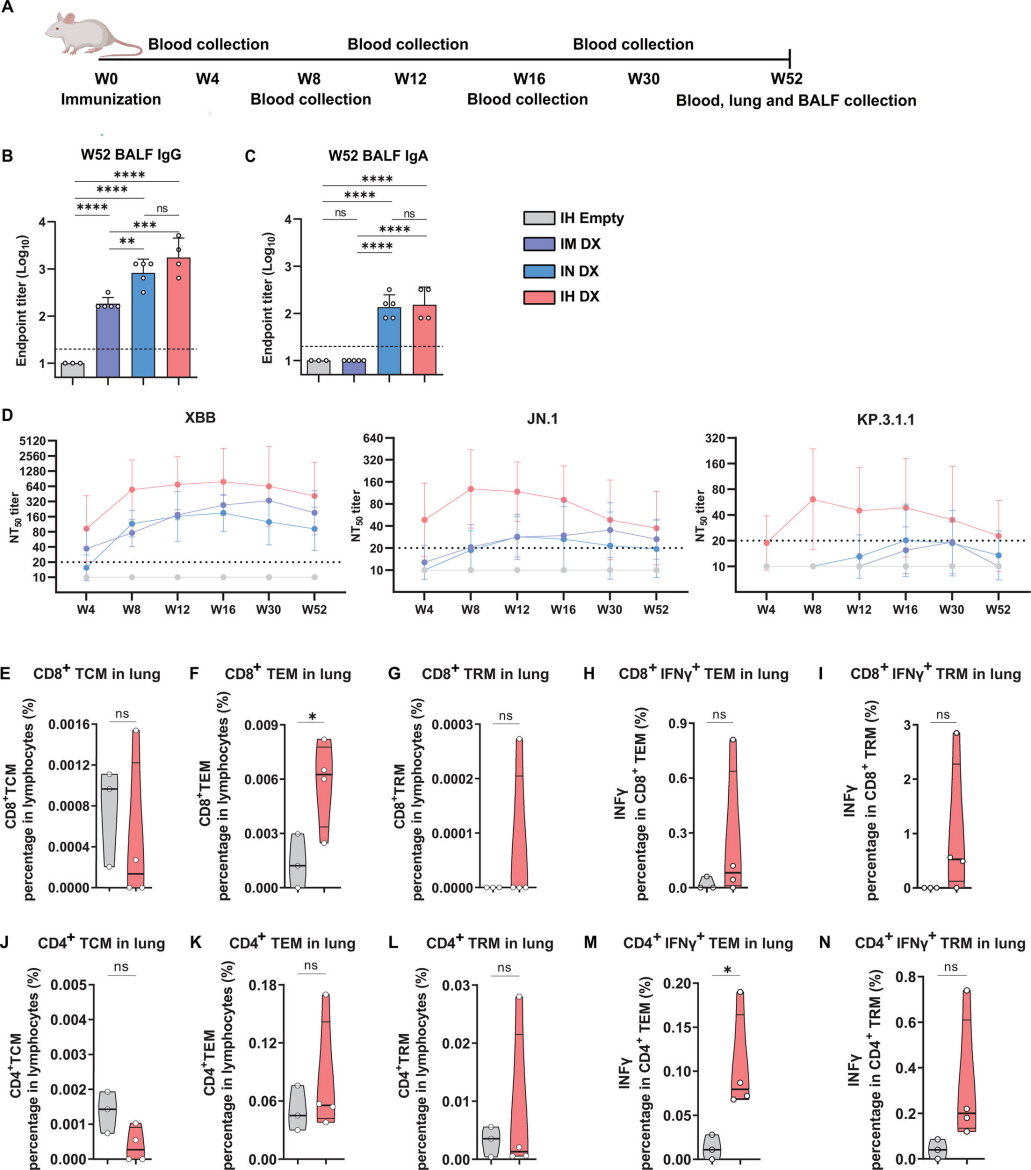
Long-term immune responses induced by AdC68-Delta-XBB. (**A**) Experimental schedule. Six- to eight-week-old female BALB/c mice (*n* = 3–5) were immunized via intramuscular injection, intranasal drip, and aerosol inhalation with AdC68-Delta-XBB. One group of mice was immunized with AdC68-Empty through aerosol inhalation as a control. Serum was collected at weeks 4, 8, 12, 16, 30, and 52 to measure antibody titers. Lung and BALF samples were collected at week 52. (**B and C**) Antibody responses in BALF at week 52 were evaluated. Anti-Delta-XBB RBD-dimer IgG (**B**) and IgA (**C**) titers were measured by ELISA. The values shown in panels **B and C** are the GMTs ± SD. The horizontal dashed lines indicate the LOD. The endpoint titer below the LOD was determined as half the LOD. Log-transformed data were statistically analyzed using ordinary one-way ANOVA, followed by Tukey’s multiple comparisons tests (ns, *P* > 0.05; **P < 0.01; ***P < 0.001; ****P < 0.0001). (**D**) Kinetics of neutralizing antibody response in murine sera. The dashed broken lines represent the pVNT_50_ for each mouse. Solid lines and circles represent geometric means for each group. The dashed horizontal lines indicate the LOD. pVNT_50_ below the LOD was determined as half the LOD. (**E–G**) The memory T cells without peptide re-stimulation were characterized by flow cytometry. The frequencies of CD8^+^ TCMs (**E**), TEMs (**F**), and TRMs (**G**) were analyzed. (**H and I**) The pneumocytes were re-stimulated with the mixed overlapping peptide pools for Delta RBD and XBB RBD and characterized by flow cytometry as CD8^+^ IFNγ^+^ TEMs (**H**) and CD8^+^ IFNγ^+^ TRMs (**I**). (**J–L**) The memory T cells without peptide re-stimulation were characterized by flow cytometry. The frequencies of CD4^+^ TCMs (**J**), TEMs (**K**), and TRMs (**L**) were analyzed. (**M and N**) The pneumocytes were re-stimulated with the mixed overlapping peptide pools for Delta RBD and XBB RBD and characterized by flow cytometry as CD4^+^ IFNγ^+^ TEMs (**M**) and CD4^+^ IFNγ^+^ TRMs (**N**). For panels **E–N**, shown are violin plots of the 25th–75th percentiles with the median as the center, and kernel density estimation curves of the distribution. Data were statistically analyzed using ordinary one-way ANOVA, followed by Tukey’s multiple comparisons tests (ns, *P* > 0.05; *P < 0.05).

To study the long-term immune responses induced by AdC68-Delta-XBB, a batch of BALB/c mice was immunized with AdC68-Delta-XBB and housed for 52 weeks (nearly 1 year) (Fig. 4A). Sera were collected at weeks 0, 4, 8, 12, 16, 30, and 52. BALF and lung samples were collected at week 52.

The results revealed that substantial IgG and IgA antibodies could be detected in BALF from the intranasal drip (GMTs of 844 and 139, respectively) and aerosol inhalation (GMTs of 1,810 and 160, respectively) groups, which were significantly higher than those detected in the intramuscular group (GMTs of 184 and 10, respectively) at 52 weeks after immunization (Fig. 4B and C). In addition, we analyzed serum neutralizing antibody kinetics against SARS-CoV-2 XBB, JN.1, and KP.3.1.1 pseudoviruses. In the intranasal and aerosol inhalation groups, the neutralizing GMTs against XBB at week 16 were 189 and 785, respectively, and declined to 92 and 412 by week 52 (Fig. 4D). In the intramuscular injection group, neutralizing antibody titers against XBB gradually increased over time and declined by week 30 (Fig. 4D). For JN.1 and KP.3.1.1, the aerosol inhalation group showed a modest rise in neutralizing antibodies by week 8, with titers gradually decreasing to near the LOD. The intramuscular and intranasal groups showed low neutralizing responses against JN.1 and KP.3.1.1, with antibody titers near or below the detection limit (Fig. 4D).

Next, we assessed pulmonary memory T-cell responses induced by aerosol inhalation of AdC68-Delta-XBB or AdC68-Empty at week 52. The percentages of TCMs, TEMs, and TRMs in CD4^+^ and CD8^+^ T cells were analyzed, as well as the IFNγ-secreting TEMs and TRMs after peptide pool stimulation (Fig. 4E through N). The results suggested that the proportions of CD8^+^ TEMs and CD4^+^ IFNγ^+^ TEMs in the AdC68-Delta-XBB group were significantly higher than those in the control group (Fig. 4F and M).

These results suggested that aerosol inhalation immunization maintained substantial antibody and cellular immune responses in mice for nearly 1 year after immunization.

### Evaluation of protective efficacy against SARS-CoV-2 infection by AdC68-Delta-XBB in a murine model

**Fig 5:**
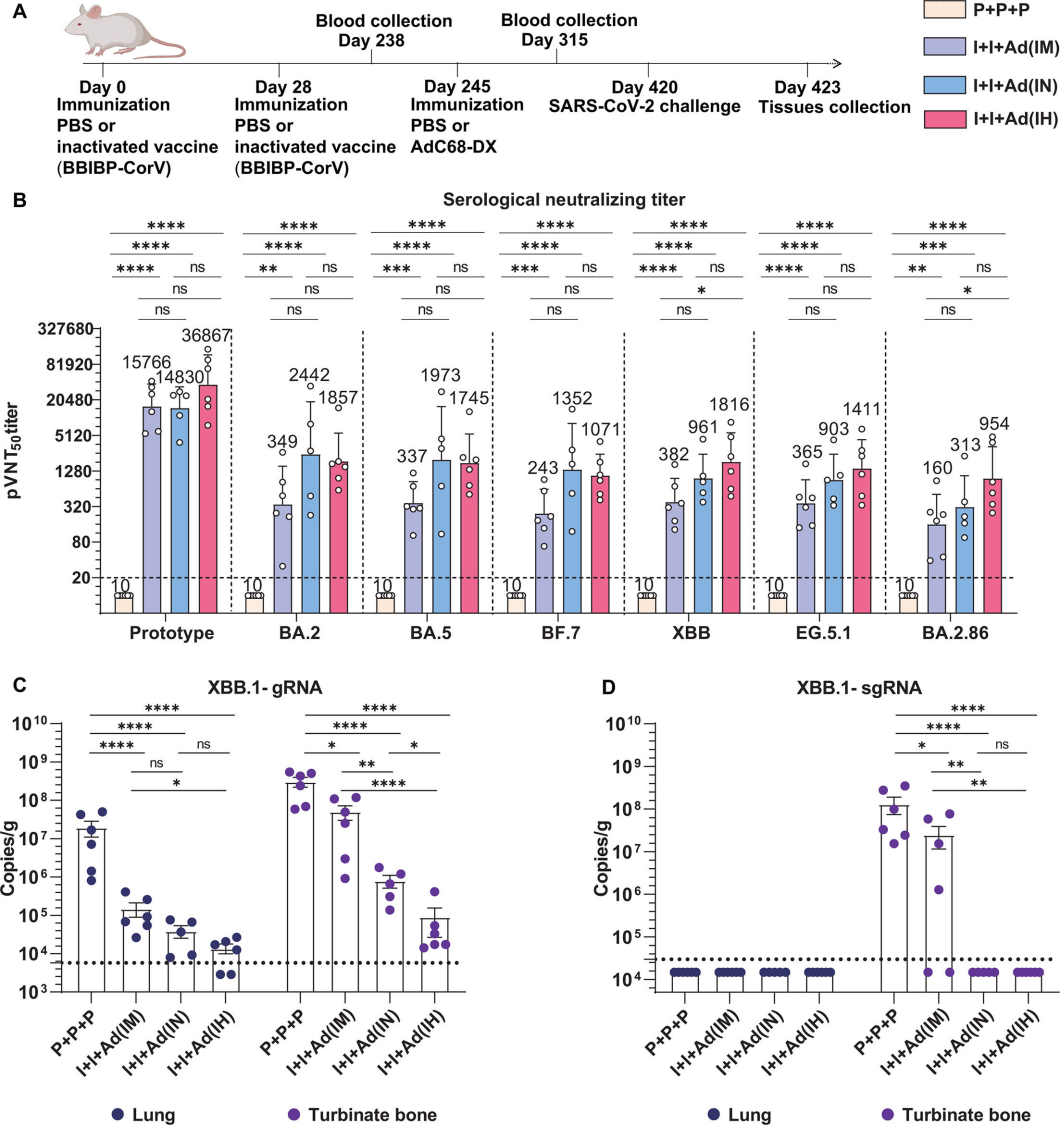
Protection efficacy of AdC68-Delta-XBB against SARS-CoV-2 infection. (**A**) Immunization and challenge schedule. Six- to eight-week-old female BALB/c mice (*n* = 5–6) were randomly divided into four groups. One group of mice received PBS as a control. The other three groups were immunized with two doses of SARS-CoV-2 inactivated vaccine (BBIBP-CorV) at days 0 and 28 and boosted with AdC68-Delta-XBB via intramuscular injection, intranasal drip, or aerosol inhalation at day 245. Serum was collected on days 238 and 315. The mice were challenged with 1 × 10^5^ TCID_50_ of SARS-CoV-2 XBB.1 via the intranasal route on day 420. Three days later, the mice were euthanized. Lung and nasal turbinate tissues were collected for viral load analysis. (**B**) Serological neutralizing titers against SARS-CoV-2 pseudoviruses. Data are GMTs ± SD. GMTs are shown at the top of each column. The dashed horizontal line indicates the LOD. pVNT_50_ below the LOD was determined as half the LOD. Log-transformed data were statistically analyzed using ordinary one-way ANOVA, followed by Tukey’s multiple comparisons tests (ns, *P* > 0.05; *P < 0.05; **P < 0.01; ***P < 0.001; ****P < 0.0001). (**C and D**) SARS-CoV-2 XBB.1 viral gRNA (**C**) and sgRNA (**D**) levels were detected by quantitative reverse transcription-PCR (qRT-PCR) in the lung and nasal turbinate tissues. The dashed horizontal lines indicate the LOD. The values are means ± SEM. Log-transformed data were statistically analyzed using ordinary one-way ANOVA, followed by Tukey’s multiple comparisons tests (ns, *P* > 0.05; *P < 0.05; **P < 0.01; ****P < 0.0001).

To investigate the protection efficacy of AdC68-Delta-XBB delivered via different immunization routes in preventing SARS-CoV-2 infection, an immunization-and-challenge experiment was conducted (Fig. 5A). A batch of BALB/c mice was sequentially immunized with COVID-19 inactivated virus vaccines and AdC68-Delta-XBB to simulate the vaccine inoculation in humans. COVID-19 inactivated virus vaccines have been widely used in China and many other countries (51–54). Groups of mice were immunized with two doses of inactivated virus vaccine (BBIBP-CorV) (55) at days 0 and 28. A booster immunization with the AdC68-Delta-XBB vaccine was then administered via intramuscular injection, intranasal drip, or aerosol inhalation on day 245 (Fig. 5A). One group of mice received three doses of PBS by aerosol inhalation as a control. Sera were collected at days 238 and 315 to measure antibody titers. Finally, the mice were challenged with SARS-CoV-2 XBB.1 on day 420. The mice were euthanized 3 days after the challenge, and lung and nasal turbinate tissues were collected for viral load analysis (Fig. 5A).

Before boosting with the AdC68-Delta-XBB vaccine, murine serum samples were collected for antibody titration. The results revealed that two doses of BBIBP-CorV induced the neutralizing antibody responses against SARS-CoV-2 prototype pseudovirus with similar GMTs (between 1,751 and 2,035) across three groups (Fig. S3). A booster of intramuscular immunization with the AdC68-Delta-XBB vaccine [I+I+Ad (IM)] induced a robust neutralizing antibody response against the prototype pseudovirus with a GMT of 15,766 (Fig. 5B). However, GMTs against Omicron sub-variants BA.2, BA.5, BF.7, XBB, and EG.5.1 were lower, ranging between 243 and 382 (Fig. 5B). In contrast, I+I+Ad (IN) and I+I+Ad (IH) groups induced more than 2.5-fold higher GMTs against Omicron BA.2, BA.5, BF.7, XBB, and EG.5.1 than the I+I+Ad (IM) group (Fig. 5B). The GMTs against BA.2.86 induced by intramuscular, intranasal, and aerosol inhalation boosting were 160, 313, and 954, respectively (Fig. 5B).

Viral load measurement revealed that the mean genomic RNA (gRNA) titer in the lungs of the control group was 1.97 × 10^7^ copies/g, while the I+I+Ad (IM) and I+I+Ad (IN) groups showed reduced mean viral loads of 1.51 × 10^5^ and 3.94 × 10^4^ copies/g, respectively (Fig. 5C). I+I+Ad (IH) showed better protective efficacy with a mean viral load of 1.38 × 10^4^ copies/g, with even 33.33% of mice exhibiting undetectable viral gRNA in the lungs (Fig. 5C). As XBB and its sub-variants primarily infect the upper respiratory tract, none of these mice showed detectable viral sub-genomic RNA (sgRNA) in the lungs (Fig. 5D).

The viral loads in the murine nasal turbinates were measured and analyzed. I+I+Ad (IM) group showed limited protection, with gRNA levels reduced by approximately sixfold compared to the control group (Fig. 5C). In contrast, the I+I+Ad (IN) and I+I+Ad (IH) groups had significantly reduced viral gRNA levels in the nasal turbinates by 381- and 3,380-fold, respectively, compared to the control group (Fig. 5C). Moreover, the nasal turbinate gRNA titer of the I+I+Ad (IH) group was significantly lower than those of the I+I+Ad (IM) and I+I+Ad (IN) groups (Fig. 5C). In addition, the mean sgRNA titer in the nasal turbinates of the control group was 1.33 × 10^8^ copies/g, and the I+I+Ad (IM) group showed a reduced viral load (5.24-folds) of 2.53 × 10^7^ copies/g (Fig. 5D). In comparison, no viral sgRNA was detected in any mice from the I+I+Ad (IN) and I+I+Ad (IH) groups, suggesting effective protection against viral infection in upper respiratory tracts (Fig. 5D). These results suggested that a booster of AdC68-Delta-XBB vaccine by aerosol inhalation provided potent protection against viral infection in both the lower and upper respiratory tracts.

## DISCUSSION

In this study, a chimpanzee AdC68-vectored SARS-CoV-2 vaccine expressing the Delta-XBB RBD-dimer was designed, and immune responses and protective efficacy induced by intramuscular injection, intranasal drip, and aerosol inhalation were comprehensively analyzed. Aerosol inhalation immunization robustly induced both humoral IgG and mucosal IgA antibodies, multifunctional T cells, and memory immune cells in the mouse lungs. Especially, BALF antibody and lung TEMs were substantially maintained 1 year after aerosol inhalation immunization, suggesting long-term immune responses. The SARS-CoV-2 XBB.1 challenge experiment demonstrated that aerosol inhalation immunization provided superior protection against upper respiratory tract viral infection compared with other IM and IN routes. These results suggest that aerosolized AdC68-Delta-XBB is a promising vaccine for preventing SARS-CoV-2 infection.

Human nasal spray vaccines, such as those for influenza (FluMist) and COVID-19 (dNS1-RBD), primarily target the upper respiratory tract (23, 56). However, the mouse model with intranasal drip immunization cannot simulate nasal spray vaccination in humans because the vaccine liquid tends to reach lung tissue when intranasally delivered to mice, unlikely to reach the upper respiratory tract in humans, thus eliciting immune responses in the lungs (57, 58). In contrast, an aerosol inhalation model was used in this study by atomizing the vaccine into micron-sized droplets, which were then inhaled by mice through both the nose and mouth. This approach better simulates the human inhalation of aerosolized vaccines via a face mask, allowing for investigation of local immune responses (19, 57, 59). However, vaccine dose is one limitation of this study. We observed that the method of vaccine inhalation in the container for a mouse led to a significant loss of aerosolized vaccine, where vaccine mainly adheres to the walls of the container tubing. To address this issue, we calculated the estimated actual dose inhaled by the mice and adjusted the aerosol dosage accordingly. For human aerosol inhalation immunization, the face mask chamber fits against the mouth. Therefore, the vaccine can be inhaled with high efficiency, allowing for a lower vaccine dose to be aerosolized (19).

The SARS-CoV-2 variants and sub-variants were classified into six serotypes based on RBD antigenicities (60–63). The XBB sub-variant was the dominant circulating sub-variant when we initiated the study. Therefore, the Delta-XBB RBD-dimer was used as the immunogen for vaccine design. However, SARS-CoV-2 continues to mutate, and the globally dominant strains are descendants of the JN.1 sub-variants (64). The World Health Organization has recommended using JN.1 and its sub-variants as vaccine antigen sequences. Although the AdC68-Delta-XBB vaccine did not contain the antigen of JN.1, as a proof-of-concept study, we demonstrated the advantages of aerosol inhalation immunization in stimulating systemic and mucosal immune responses, and the vaccine antigen sequence can be quickly updated in preclinical studies according to currently circulating strains. This is another limitation of this study.

Another study found that a bivalent Ad26-based SARS-CoV-2 vaccine boosted via the intratracheal route induced mucosal neutralizing antibodies, IgG and IgA binding antibodies, and CD8^+^ and CD4^+^ T cell responses in macaques, conferring robust protection against SARS-CoV-2 BQ.1.1 challenge (33). Results from our study here demonstrated that aerosolized AdC68-Delta-XBB vaccine elicited robust antibody and T-cell immune responses in the lower respiratory tract (lung). The aerosolized vaccine could reach both the upper and lower respiratory tracts of mice during inhalation immunization, resulting in the inference that the vaccine induces immune responses in the upper respiratory tract, thereby reducing susceptibility to viral infection in the noses of mice. Therefore, the aerosol inhalation immunization with AdC68-Delta-XBB vaccine prevented nasal infection by SARS-CoV-2 XBB.1 in our challenge experiment.

This study evaluated the immune responses and protective efficacy elicited by intramuscular injection, intranasal drip, or aerosol inhalation in a mouse model, demonstrating that aerosol inhalation of AdC68-Delta-XBB offers a distinct advantage in robustly stimulating both systemic and mucosal immune responses. These findings provide valuable insights for developing mucosal vaccines targeting other respiratory pathogens.

## MATERIALS AND METHODS

### AdC68-Delta-XBB vaccine

SARS-CoV-2 Delta-XBB RBD-dimer was one Delta RBD (S protein residues 319–537, GenBank: OK091006.1) and one Omicron XBB RBD (S protein residues 316–534, GISAID: EPI_ISL_16392105) connected as a tandem repeat (40, 44). The Delta-XBB RBD-dimer gene was inserted into the E1-deleted region of the replication-deficient chimpanzee adenoviral vector AdC68. AdC68-Empty was employed as a control, with no insertion at the E1-deleted locus. Recombinant adenovirus was rescued and propagated in HEK293 cells and purified by CsCl gradient ultracentrifugation, as previously described (42). Viral particles were calculated using spectrophotometry.

### Western blot

HEK293T cells were pre-plated in six-well plates, followed by infection with AdC68-Delta-XBB or AdC68-Empty as a control at a dose of 1 × 10^11^ vp. Forty-eight hours post-infection, culture supernatants were collected. Protein samples were separated by 12% SDS-polyacrylamide gel electrophoresis and analyzed by western blot with rabbit anti-RBD SARS-CoV-2 polyclonal antibodies. Goat anti-rabbit IgG-horseradish peroxidase (HRP) antibody was used as a secondary antibody. The membranes were developed using the SuperSignal West Pico chemiluminescent substrate (Thermo Fisher Scientific).

### Measurement of the inhaled dose of aerosolized AdC68-Delta-XBB vaccine

The entire respiratory tract of mice was collected 2 h after immunization. Tissues were placed in tubes containing 500 μL of PBS, homogenized, and centrifuged. Viral DNA was then extracted from 50 μL of the supernatant using a MagMAX Express Magnetic Particle Processor according to the manufacturer’s instructions. AdC68-specific real-time PCR (RT-PCR) assays were performed by using a SuperReal PreMix (Probe) kit (Tiangen Biotech) on a CFX96 Touch real-time PCR detection system (Bio-Rad) according to the manufacturer’s protocol. The sets of primers and probe were used to detect the gDNA with sequences as follows: gDNA-F, CAGCTGAATGCTGTGGTTGA; gDNA-R, CACCGCCTGATTCCACATAC; gDNA-probe, FAM-ACCGAGCTGTCCTACCAGCTCTTGCT-TAMRA (where FAM is 6-carboxyfluorescein and TAMRA is 6-carboxytetramethylrhodamine).

Viral loads were expressed on a log10 scale as viral copies/g after calculation with a standard curve. Viral copy numbers below the limit of detection were set as half of the LOD.

### Mouse experiments

All animals in this work were specific pathogen-free female BALB/c mice (Beijing Vital River Laboratory Animal Technology Co., Ltd.). AdC68-Delta-XBB and AdC68-Empty were diluted in PBS. AdC68-Empty and PBS were used as controls. Female BALB/c mice at 6–8 weeks of age were immunized with vaccine through intramuscular injection, intranasal drip, or aerosolized inhalation. Intramuscular injection immunizations were performed with 2.5 × 10^10^ vp vaccines (100 μL). Intranasal drip immunizations were performed with 2.5 × 10^10^ vp vaccines (50 μL). Aerosolized inhalation immunizations were performed with 2.5 × 10^12^ vp vaccines (400 μL) (animal oral and nasal exposure device, Shanghai Yuyan Instruments Co., Ltd., S1001M). Mice were immunized intramuscularly with BBIBP-CorV (1.3 U/mice), 1/5 of the human dose.

For the challenge experiment, the mice were intranasally infected with 1 × 10^5^ TCID_50_ of SARS-CoV-2 (XBB.1, NPRC 2.192100020). Three days post-challenge, mice were euthanized and necropsied. Lungs and nasal turbinate tissues were collected. All animal experiments with SARS-CoV-2 were conducted under the animal biosafety level 3 (ABSL-3) facility at the Chinese Center for Disease Control and Prevention (China CDC).

### Enzyme-linked immunosorbent assay

Enzyme-linked immunosorbent assays (65) were conducted to measure the antigen-specific antibody titers. Briefly, 96-well plates were coated with 3 μg/mL of Delta-XBB RBD-dimer protein at 4°C overnight and blocked with 5% skim milk in PBST (0.05% Tween 20 in PBS) at 37°C for 1 h. The plates were incubated with diluted murine serum or BALF for 2 h at 37°C. Then, the plates were incubated with goat anti-mouse IgG-HRP antibody (Abcam) or goat anti-mouse IgA-HRP antibody (Abcam) and developed with 3,3′,5,5′-tetramethylbenzidine (TMB) substrate. Reactions were stopped with 2 M hydrochloric acid, and the absorbance was measured at 450 nm using a microplate reader (PerkinElmer). The endpoint titers were defined as the highest reciprocal dilution of samples to give an absorbance greater than 2.5-fold of the background values. The antibody titer below the LOD was determined as half the LOD.

### Enzyme-linked immunospots

Murine lungs and spleens were harvested. Splenocytes and pneumocytes were isolated. Then, the ELISpot Mouse IgG (HRP) kit (MABTECH) and ELISpot Mouse IgA (HRP) kit (MABTECH) were used. The plates were pre-coated with 15 μg/mL of Delta-XBB RBD-dimer protein overnight at 4°C, washed with sterile PBS, and then blocked. Single-cell suspensions of splenocytes or pneumocytes were added to the plates and incubated at 37°C for 24 h. After incubation, the cells were removed and washed with PBS. The plates were then incubated with biotinylated anti-IgG or anti-IgA, followed by streptavidin-conjugated HRP and developed with TMB substrate solution according to the kit guidelines (MABTECH). The development was stopped by thoroughly rinsing samples with deionized water. The number of spots was determined using an automatic ELISpot reader (Cellular Technology Ltd.).

### Pseudovirus neutralization assay

SARS-CoV-2 pseudovirus generation was performed as previously described (61). Briefly, the spike protein with 18 amino acids deleted from the C-terminus of the SARS-CoV-2 prototype and the codon-optimized spike protein of each Omicron sub-variant were cloned into the pCAGGS vector, respectively. A total of 30 μg of each construct was transfected into HEK-293T cells. VSV-ΔG-GFP pseudotyped virus was added 24 h after transfection. After 2 h of infection, the VSV-ΔG-GFP residues were removed by changing the medium to fresh complete DMEM containing anti-VSV-G antibodies (I1HybridomaATCC-CRL2700). After incubation at 37°C for 30 h, the supernatants were collected, filtered through a 0.45 μm filter (Millipore), and then aliquoted and stored at −80°C.

The heat-inactivated (56°C for 30 min) murine sera were twofold serially diluted and incubated with pseudoviruses at 37°C for 1 h. Then, the mixture was transferred to pre-plated Vero-E6 cells in 96-well plates. After incubation for 15 h, the transducing unit numbers were calculated on a CQ1 confocal image cytometer (Yokogawa). The pVNT_50_ was determined by fitting nonlinear regression curves using GraphPad Prism and calculating the reciprocal of the serum dilution required for 50% neutralization of infection. pVNT_50_ below the LOD was determined as half the LOD.

### Flow cytometry

To analyze the memory T-cell responses, the murine pneumocytes were stained with anti-CD3 (BioLegend), anti-CD4 (BioLegend), anti-CD8α (BioLegend), anti-CD44 (BioLegend), anti-CD62L (BioLegend), and anti-CD69 (BioLegend) antibodies. TCMs were gated with CD44^+^ CD62L^+^ T cells. TEMs were gated with CD44^+^ CD62L^−^ T cells. TRMs were gated with CD44^+^ CD62L^−^ CD69^+^ T cells. To analyze IFNγ secretion in different memory T cells, the murine pneumocytes were stimulated with the mixed SARS-CoV-2 Delta and XBB RBDs overlapping peptide pool (2 μg/mL) and simultaneously treated with GolgiStop (BD Biosciences). After 12 h of incubation, the cells were harvested and stained with anti-CD3 (BioLegend), anti-CD4 (BioLegend), anti-CD8α (BioLegend), anti-CD44 (BioLegend), anti-CD62L (BioLegend), and anti-CD69 (BioLegend) surface markers. The cells were subsequently fixed, permeabilized in permeabilizing buffer (BD Biosciences), and stained with anti-IFNγ (BioLegend) antibodies. To analyze the cytokine secretion, the murine pneumocytes were stimulated with the mixed SARS-CoV-2 Delta and XBB RBDs overlapping peptide pool (2 μg/mL) and simultaneously treated with GolgiStop (BD Biosciences). After 12 h of incubation, the cells were harvested and stained with anti-CD3 (BioLegend), anti-CD4 (BioLegend), and anti-CD8α (BioLegend) surface markers. The cells were subsequently fixed, permeabilized in permeabilizing buffer (BD Biosciences), and stained with anti-IFNγ (BioLegend), anti-TNFα (BioLegend), anti-IL-2 (BioLegend), and anti-IL-4 (BioLegend) antibodies. All fluorescent lymphocytes were analyzed on a BD LSRFortessa flow cytometer (BD Biosciences).

The gating strategy used to identify antigen-specific cytokine-secreting T cells, TCMs, TEMs, and TRMs is illustrated in Fig. S4 and S6.

As shown in Fig. S4, the frequency of antigen-specific T cells was calculated as the percentage of cytokine-positive cells within the parent CD8^+^ or CD4^+^ T cells. For Boolean gating analysis of monofunctional, bifunctional, and trifunctional (IFNγ^+^, TNFα^+^, and IL-2^+^) cells, data are expressed as the percentage of each functional subset within the total CD8^+^ or CD4^+^ T cell populations.

As shown in Fig. S6, the frequencies of TCMs, TEMs, and TRMs were calculated as percentages of the total lymphocyte population. The frequencies of IFNγ^+^ TEMs and IFNγ^+^ TRMs were calculated relative to the TEM and TRM populations, respectively. To further define the composition of these IFNγ^+^ T cell populations, TRMs and other T cells were quantified as percentages of the total CD8^+^ or CD4^+^ T cells.

### Quantitative reverse transcription PCR

Mouse lung and nasal turbinate tissues were weighed and homogenized. Viral RNA was isolated from 50 μL of supernatants of homogenized tissues using a nucleic acid extraction instrument, MagMAX Express Magnetic Particle Processor (Applied Biosystems). SARS-CoV-2-specific quantitative reverse transcription-PCR (qRT-PCR) assays were performed using a FastKing One Step Probe RT-qPCR kit (Tiangen Biotech) on a CFX96 Touch real-time PCR detection system (Bio-Rad) according to the manufacturer’s protocol. Two sets of primers and probes were used to detect the viral gRNA and sgRNA with sequences as follows: gRNA-F, GACCCCAAAACCAGCGAAAT; gRNA-R, TCTGGTTACTGCCAGTTGAATCTG; gRNA-probe, FAM-ACTCCGCATTACGTTTGGTGGACC-TAMRA; sgRNA-F, TGATCTCTTGTAGATCTGTTCTC; sgRNA-R, ATATTGCAGCAGTACGCACACA; sgRNA-probe, FAM-ACACTAGCCATCCTTACTGCGCTTCG-TAMRA.

Viral loads were expressed on a log_10_ scale as viral copies/g after calculation with a standard curve. Viral copy numbers below the LOD were set as half of the LOD.

### Statistical analysis

The statistical analyses were performed by GraphPad Prism 10.1 for all experiments. The statistical details of comparisons can be found in the figures and figure legends.

## Data Availability

All study data are included in the main text and supplemental material.

## SUPPLEMENTAL MATERIAL

Supplemental figures mbio.01464-26-s0001.docx Fig. S1 to S6.

ASM does not own the copyrights to Supplemental Material that may be linked to, or accessed through, an article. The authors have granted ASM a non-exclusive, world-wide license to publish the Supplemental Material files. Please contact the corresponding author directly for reuse.
