# Supplementary material for: Mucosal boosting of chimpanzee adenovirus-vectored vaccine encoding SARS-CoV-2 RBD-heterodimer provides robust protection in mice
Source: mBio. 2026 Jul 21;17(8):e01464-26. doi: 10.1128/mbio.01464-26 (PMC13463698; doi:10.1128/mbio.01464-26)
Supplement: Supplementary file 1 [file mbio.01464-26-s0001.docx]

Supporting Information for

Mucosal boosting of Chimpanzee adenovirus-vectored vaccine encoding SARS-CoV-2 RBD-heterodimer provides robust protection in mice

Xueyuan Liu, Yaling An, Huixin Duan, Fei Shao, Ke Xu, Chenxi Yang, Senyu Xu, Xuemei Zhou, Minrun Duan, Tianyi Zheng, Shiyu Sun, Mengjie Yang, Kun Cai, William J. Liu, Dongming Zhou, Kun Xu, Lianpan Dai, Shuo Wang, Peipei Liu, Wei Ma, George F. Gao

*Correspondence: Shuo Wang, Peipei Liu, Wei Ma, George F. Gao

Email: [wangshuo@im.ac.cn](mailto:wangshuo@im.ac.cn) (S.W.); [liupp@ivdc.chinacdc.cn](mailto:liupp@ivdc.chinacdc.cn) (P.L.); weima@sdu.edu.cn (W.M.); [gaof@im.ac.cn](mailto:gaof@im.ac.cn) (G.F.G.)

# This file includes:

Figures S1 to S6

**kDa**

**AdC68-Empty**

**AdC68-DX**

**180**

**140**

**100**

**75**

**60**

**45**

**35**

**25**

**15**

**10**

# Figure S1. Antigen expression of the AdC68-Delta-XBB vaccine.

HEK293T cells were infected with AdC68-Delta-XBB or AdC68-Empty (control). Antigen protein was detected in cell supernatants by Western blot.

**gDNA**

ns

✱✱✱✱

**10^8^**

✱✱✱✱

**10^7^**

**10^6^**

**10^5^**

**Copies/g**

**10^4^**

**10^3^**

**10^2^**

**10^1^**

**10^0^**

**IN 2.5×10**

10

**vp**

**IH 2.5×10**

10

**vp**

**IH 2.5×10**

11

**vp**

**IH 2.5×10**

12

**vp**

# Figure S2. Titration of the inhaled dose of aerosolized AdC68-Delta-XBB vaccine in a BALB/c mouse model.

Levels of AdC68 gDNA from mixed murine lung, trachea and nasal turbinate tissues were measured by quantitative PCR. The values are means ± SEM. Log-transformed data were statistically analyzed using ordinary one-way ANOVA followed by Tukey's multiple comparisons tests (ns, p > 0.05; ****p < 0.0001).

**Serological neutralizing titer (Day 238) 20480**

2035 1751 1984

10

**5120**

**1280**

**pVNT_50_ titer**

**320**

**80**

**20**

**P+P+P**

**I+I+Ad(IN)**

**I+I+Ad(IH)**

**I+I+Ad(IM)**

**Figure S3**. **Serological neutralizing titer against prototype SARS-CoV-2 pseudovirus.** Murine sera were collected on day 238. The pVNT_50_ against prototype SARS-CoV-2 pseudovirus were measured. Data are GMTs ± SD. GMTs are shown at the top of each column. The dashed horizontal line indicates the LOD. pVNT_50_ below the LOD was determined as half the LOD.

**Figure S4**. **Gating strategy for T cells, related to Fig 2.**

**Figure S5**. **Percentages of T-cell subsets, related to Figs. 2K, 2L, 3L and 3M.**

(A) Average frequency of monofunctional, bifunctional, and trifunctional cells among CD8^+^ T cells (related to Fig. 2K).

(B) Average frequency of monofunctional, bifunctional, and trifunctional cells among CD4^+^ T cells (related to Fig. 2L).

(C) Average frequency of IFNγ-producing TRMs among total CD8^+^ T cells (related to Fig. 3L).

(D) Average frequency of IFNγ-producing TRMs among total CD4^+^ T cells (related to Fig. 3M).

**Figure S6**. **Gating strategy for T cells, related to Fig 3.**

Figs. 3A–K were generated based on the lymphocyte populations gated using the strategy in the blue box. Figs. 3L and 3M were generated based on the functional and phenotypic analyses gated using the strategy in the red box. Specifically, these charts display the proportion of TRMs within the IFNγ⁺ T cell population.
